# Anti‐Inflammatory Efficacy of a Functional Shampoo Mixed With 0.063% Artemether for Seborrheic Dermatitis

**DOI:** 10.1111/jocd.70540

**Published:** 2025-11-13

**Authors:** Yuqian Song, Mengyi Feng, Yiwei Gao, Genong Sun, Chenyu Zhao

**Affiliations:** ^1^ Department of China Medical University‐The Queen's University of Belfast Joint College, School of Pharmacy China Medical University Shenyang China; ^2^ School of Pharmacy Queen's University Belfast Belfast UK; ^3^ State Key Laboratory of Discovery and Utilization of Functional Components in Traditional Chinese Medicine, Key Laboratory of Chemical Biology (Ministry of Education), Institute of Pharmacognosy, School of Pharmaceutical Sciences Shandong University Jinan China

**Keywords:** artemether, functional shampoo, seborrheic dermatitis

To the Editor,

We have read with interest the recent article by Pei et al., “Anti‐Inflammatory Efficacy of a Functional Shampoo Mixed With 0.063% Artemether for Seborrheic Dermatitis” [1], and we commend the authors' efforts to explore novel therapeutic strategies for the common condition of seborrheic dermatitis (SD). However, upon in‐depth review of the study's methodology and conclusions, we have identified several fundamental methodological and logical flaws that severely undermine the reliability of its core conclusions. This letter aims to systematically discuss three interconnected critical issues: the limited clinical relevance of the chosen animal model, the unexplained negative results of the positive control group, and a resultant paradox that is sufficient to challenge the study's entire foundation. We also wish to offer our perspective on future research and the clinical translation of these findings.

The study employed an acute inflammatory model induced by the combined application of olive oil and *Malassezia furfur* on the dorsal skin of guinea pigs. However, a significant chasm exists between this model at the pathophysiological level and the complexity of human SD. Human SD is a quintessential chronic, recurrent disease, with a pathogenesis driven by the interplay of three core factors: colonization by *Malassezia* species (primarily *M. restricta* and *M. globosa* ), abnormal sebum production, and individual host susceptibility, which includes immune dysregulation and skin barrier dysfunction [2]. The model constructed by Pei et al. is, in essence, an exogenously induced acute contact dermatitis model. It simulates a one‐time “acute attack” rather than a “chronic disease state” driven by intrinsic immune imbalance. This model fails to recapitulate the chronic course, relapsing nature, complex T‐cell‐mediated immune disturbances, and long‐term impairment of the skin barrier that characterize human SD [3]. Consequently, the study's findings merely reflect the capacity of artemether to suppress an acute, artificially induced inflammation over a short period, while providing virtually no information on its potential efficacy in managing a chronic disease. This disconnect between the model and clinical reality fundamentally limits the external validity and translational value of the study's findings [4].

The most damaging finding in this study is the observation that the positive control, 2% ketoconazole shampoo, while clinically effective, failed to significantly downregulate the expression levels of interleukin‐1β (IL‐1β), nuclear factor‐κB (NF‐κB), and the histamine H_1_ receptor in lesional tissue. Rather than questioning this anomalous result, which contradicts established pharmacological knowledge, the authors used it as a cornerstone to build their central conclusion that “artemether may exhibit stronger anti‐inflammatory effects”. This represents a fundamental logical fallacy. A substantial body of scientific literature has firmly established that ketoconazole, a first‐line therapy for SD, derives its efficacy not only from its antifungal activity but also from its independent and significant direct anti‐inflammatory properties [5, 6, 7]. Ketoconazole is known to inhibit the 5‐lipoxygenase pathway, thereby reducing the production of potent pro‐inflammatory leukotrienes, and possesses broad immunosuppressive activity [8].

Therefore, the “failure” of the ketoconazole group does not imply an absence of anti‐inflammatory activity. Instead, it strongly points to a more profound issue: the experimental system employed in the study (i.e., the *M. furfur*‐induced guinea pig model and its associated experimental conditions) lacks the sensitivity and specificity to detect the known pharmacological effects of a validated therapeutic agent. If an experimental platform fails to generate the expected positive signal for a gold‐standard positive control, its reliability in assessing the response to a novel, unproven agent like artemether is rendered highly questionable. The failure of the positive control acts as an alarm, signaling a potential systemic flaw in the entire experimental platform. Any comparative conclusion drawn based on a “failed” positive control is thereby scientifically invalidated.

The aforementioned paradox is closely linked to another major omission in the study's design: the absence of a justification for sample size and statistical power. The sample size of 10 animals per group was set without any a priori power analysis. In preclinical research, power analysis is an indispensable step to ensure the reliability of results and to avoid Type II errors (i.e., false negatives).

Due to the lack of a power analysis, it is impossible to discern whether the negative results in the ketoconazole group reflect a genuine lack of effect in this specific model or are a consequence of an insufficient sample size, which would render the study underpowered to detect a true existing anti‐inflammatory effect. Without being able to exclude the latter possibility, the authors' conclusion that “artemether has a stronger anti‐inflammatory effect,” based on the observation that “ketoconazole was ineffective while artemether was effective,” is logically untenable. The cornerstone of this core conclusion—the “failure” of the positive control group—is likely an artifact of intrinsic flaws in the study design rather than a true reflection of the drug's properties.

In summary, an animal model severely disconnected from clinical reality has produced a paradoxical result for the positive control that contradicts established scientific knowledge, a result which itself cannot be properly interpreted due to a lack of statistical power analysis. These three points collectively lead to the conclusion that the data currently presented are far from sufficient to support the study's central claims, and certainly do not provide an adequate basis for advancing a 0.063% artemether shampoo into human clinical trials. To genuinely assess the therapeutic potential of artemether in SD, future research must be built upon a more robust scientific foundation. This necessitates the development of a more clinically relevant preclinical research blueprint, where future animal experiments should endeavor to use models that better simulate the chronic, immune‐dysregulated characteristics of human SD, such as certain genetically engineered mouse models or models employing long‐term colonization with clinically relevant species like *M. restricta*.

The study duration should be appropriately extended and include a posttreatment follow‐up period to evaluate long‐term efficacy and recurrence rates. Furthermore, the assessment of outcomes should not be confined to clinical scores and semiquantitative immunohistochemical analysis of a few inflammatory factors. Instead, it should incorporate more objective and comprehensive measurement dimensions, such as the direct instrumental measurement of transepidermal water loss (TEWL) to assess skin barrier function, direct quantification of fungal burden via qPCR or culture, and a more comprehensive analysis of inflammatory pathway changes through techniques like transcriptomics [9, 10]. Finally, all preclinical studies must be underpinned by rigorous study design and statistical analysis, including scientific sample size estimation and appropriate statistical treatment of data, to ensure the robustness of the conclusions.

We believe that only through such rigorous, multidimensional preclinical research can a solid chain of evidence be constructed for a potential new therapeutic agent. This is the only path that can pave the way for well‐designed, patient‐centric clinical trials and, ultimately, offer a genuine prospect of relief for patients suffering from SD.

## Author Contributions

All authors extensively discussed the original manuscript. Y.S. and C.Z. conceptualized and wrote the letter. M.F. and Y.G. provided information for this letter and edited the letter to final version. All authors reviewed and approved the final version of the manuscript.

## Ethics Statement

The authors have nothing to report.

## Conflicts of Interest

The authors declare no conflicts of interest.

## Data Availability

Data sharing not applicable to this article as no datasets were generated or analyzed during the current study.
